# Progressive Model-Driven Approach for 3D Modeling of Indoor Spaces

**DOI:** 10.3390/s23135934

**Published:** 2023-06-26

**Authors:** Ali Abdollahi, Hossein Arefi, Shirin Malihi, Mehdi Maboudi

**Affiliations:** 1 School of Engineering, Faculty of Surveying and Geospatial Engineering, University of Tehran, Tehran 1417614411, Iran; aliabdollahi@ut.ac.ir (A.A.); hossein.arefi@hs-mainz.de (H.A.); 2 Department of Geoinformatics and Surveying, School of Engineering, Mainz University of Applied Sciences, 55128 Mainz, Germany; 3 School of Engineering, University of Edinburgh, Edinburgh EH9 3JL, UK; smalihi@ed.ac.uk; 4 Institute of Geodesy and Photogrammetry, Technische Universität Braunschweig, 38106 Braunschweig, Germany

**Keywords:** 3D indoor modeling, 3D reconstruction, model-driven, point cloud, laser scanning, BIM

## Abstract

This paper focuses on the 3D modeling of the interior spaces of buildings. Three-dimensional point clouds from laser scanners can be considered the most widely used data for 3D indoor modeling. Therefore, the walls, ceiling and floor are extracted as the main structural fabric and reconstructed. In this paper, a method is presented to tackle the problems related to the data including obstruction, clutter and noise. This method reconstructs indoor space in a model-driven approach using watertight predefined models. Employing the two-step implementation of this process, the algorithm is able to model non-rectangular spaces with an even number of sides. Afterwards, an “improvement” process increases the level of details by modeling the intrusion and protrusion of the model. The 3D model is formed by extrusion from 2D to 3D. The proposed model-driven algorithm is evaluated with four benchmark real-world datasets. The efficacy of the proposed method is proved by the range of [77%, 95%], [85%, 97%] and [1.7 cm, 2.4 cm] values of completeness, correctness and geometric accuracy, respectively.

## 1. Introduction

The automatic or semi-automatic reconstruction of 3D indoor models of buildings is of great interest in photogrammetry, computer vision and computer graphics for its wide applications in construction management, emergency situations and management of natural or unnatural crises [1,2]. Due to challenges such as architectural diversity and the presence of obstructive furniture, gaps and noise in the data, the topic of “3D modeling of indoor spaces” has significant importance in current research [3]. Laser scanners are the most common solution for 3D data acquisition in indoor environments [4]. Stationary laser scanners collect points from one or more stations in a 3D coordinate system, while indoor mobile laser scanners (IMMS), mainly based on SLAM, collect data at a given rate by moving the sensor. In indoor environments, where there are usually people and furniture in the room, it is easier and faster to use IMMS.

**Figure 1:**
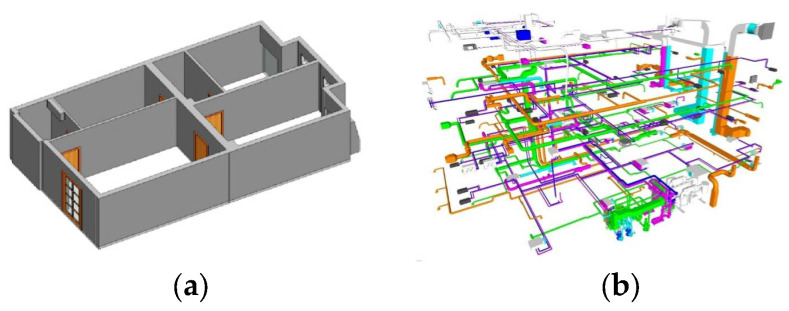
Different types of BIM: (**a**) An example of a 3D model of structural elements (architectural BIM), Reprinted with permission from Ref. [8]. 2023, “Elsevier”. (**b**) An example of a 3D model of a building’s mechanical facilities (adopted from srinsofttech.com, accessed on 23 August 2022).

Over the last 5 years, the number of papers presented on building information modeling (BIM) have increased exponentially [5]. BIM is not limited to modeling the main structural elements of a building, but can also include the mechanical, electrical and plumbing systems of the building (Figure 1) [6,7]. The reliable outputs of the automatic reconstruction of 3D building models, such as 3D coordinates of interior vertices, can be a suitable input for BIM [8].

The most fundamental challenges in automatic 3D indoor modeling include the presence of gaps and noise as well as the presence of furniture in the point cloud [9]. Therefore, the use of classical data-driven methods [10] may not always be a suitable solution to the problem of 3D indoor modeling. This motivates us to research and develop model-driven methods. In data-driven methods, a 3D model is obtained by extracting and reconstructing structural elements and connecting them, while in model-driven methods, the final 3D model is obtained from an approximate initial model from predefined libraries. Therefore, our main goal in this study is to provide a model-driven method that can reduce the negative effects caused by local gaps, noise and clutter in the point cloud. The proposed 3D reconstruction method, which is a progressive model-driven approach, can reduce the challenges of data-driven methods because by determining an initial model as a framework, problems such as partial gaps, undiscovered sides or additional sides can be decreased. Also, in our proposed method, there are no problems caused by the intersection of the planes, and the output would be a water-tight 3D model of the indoor spaces.

This paper is structured as follows: In Section 2, state-of-the-art methods are discussed. Section 3 describes the proposed model-driven method. The experiments and results on real-world benchmark datasets, as well as the comparison with state-of-the-art methods, are presented in Section 4. Section 5 concludes the paper with a discussion of the proposed method and recommendations for future work.

## 2. Related Work

The first research and efforts in 3D indoor modeling can be traced back to 2000 [3]. Since 2010, there has been more attention and a remarkable increase in the number of proposals of approaches [3]. Furthermore, since 2016, more innovative approaches and remarkable results are being seen in the research papers. Indoor modeling strategies can be classified into three main categories: data-driven, model-driven and hybrid or procedural methods.

Most methods use the point cloud as the main data. Typically, IMMS or stationary laser scanners are used for data acquisition [4]. In addition, some studies use auxiliary data along with the point cloud to improve the approach. Some of the auxiliary data are the coordinates of the positions of the laser scanners to help detect occluded areas [11] or to separate different rooms of a building [12]. Another example of widely used auxiliary data are the trajectories of the IMMS. In various studies, these data are used to detect occluded areas of a point cloud using ray tracing [13] to separate different floors in multi-floor data, and to detect the approximate location of doors [14].

Traditional data-driven methods: Data-driven methods can be divided into two categories: traditional data-driven methods [1,7,15] and graph optimization data-driven methods [12,16,17]. The main objective of traditional data-driven methods is to extract the planes of the structural elements (walls, ceiling and floor) of building interiors in different ways, and by intersecting these extracted planes, a 3D model is reconstructed. Plane extraction in these methods is usually conducted via RANSAC-based plane detection [6,9,10], or by using boundary tracing and boundary extraction methods on segmented point clouds [18,19]. These extracted planes or patches are then intersected automatically [17,19] or semi-automatically [20]. The most fundamental challenges of these methods are noise, gaps and interior furniture, which cause some problems in the surface extraction process [2]. As a result, they can lead to unrealistic anomalies in the final 3D model. In order to control such problems in the model, some researchers add an enhancement stage based on the rules governing the interior space, which can improve some of these anomalies according to the data conditions [9,15,18].

Graph optimization data-driven methods: Following the problems of automatic plane intersection based on predefined rules, graph-based data methods have been developed to increase the automation of the procedure. In these methods, after extracting the main structural planes, a 2D [20] or 3D cell/graph decomposition [16,17] is usually performed, taking into account all plane intersection probabilities. A cost function is then defined based on the probability rules and existing data conditions, and optimization is usually performed in an iterative process.

**Figure 2:**
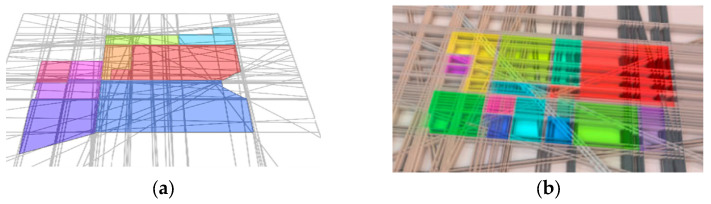
Graph optimization examples (Colors shows separate closed spaces): (**a**) 2D graph optimization, Reprinted with permission from Ref. [12]. 2023, “Elsevier”, (**b**) 3D graph optimization, Reprinted with permission from Ref. [16]. 2023, “Elsevier”.

After optimization, it is determined whether each cell is full or empty, and the same label is assigned to all cells belonging to the same room (Figure 2). Graph-based methods are more robust to imprecision and incompleteness of the point cloud compared to traditional methods, but the algorithms are usually based on the extraction of structural planes, which can be disturbed by the presence of gaps in the point cloud [2].

Model-driven method: The number of presented model-driven methods is very limited to our knowledge. Shape grammar [21] is one of the successful model-driven methods. In this case, a basic shape is defined with six parameters (three dimensions + three transitions). Considering that the data have already been aligned with the main walls, the rotation parameters are omitted. In this process, the initial model is transformed into the final model after the sequential application of six topological rules in the form of a chain process and with a certain number of repetitions. The authors consider the limitation to the world of Manhattan and the manual modeling of building doors to be one of the fundamental problems. They are also looking for ways to reduce the sensitivity of the method to gaps and higher amounts of interior furniture.

Hybrid (procedural) methods: The combination of data-driven and model-driven views can cover each other’s challenges. The authors of [21] used shape grammar and eight topological rules with an rjMCMC algorithm to create a 3D model [2]. In this hybrid method, the limitations of Manhattan modeling were overcome and the modeling of doors and windows was performed manually. The authors also aim to use a method with better performance in extracting structural planes in large and complex buildings in their future work.

Reconstruction can be volumetric [8,16] or surface [11,17], in the sense that the space between the walls of adjacent rooms is empty or volumetrically full. In volumetric reconstruction, the thickness of the partition walls is calculated and the thickness of the exterior walls is determined with prior knowledge of the environment [8,21]. It should be noted that in the BIM, standard walls are represented as volumetric objects, but in the CityGML standard, the reconstruction is planar [22].

**Figure 3:**
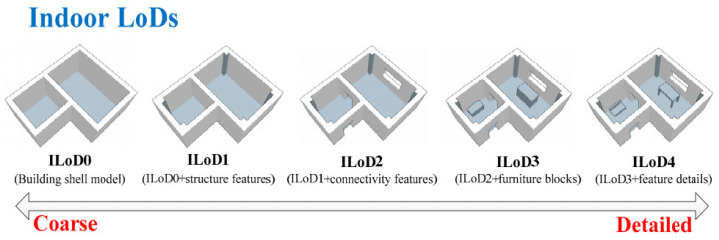
Indoor spaces LoD definition [27].

The reconstruction of the 3D model can be conducted simultaneously [2,9], or after labeling the interior spaces [15,18,23]. In some recent studies, the segmentation of spaces was discussed separately [4,24,25]. Different methods have been presented for the separation of interior spaces. The majority of these methods are based on morphological operators in the 2D space [24] or in the 3D space [26]. Room segmentation adds useful semantic information to start the 3D modeling process, depending on the approach used [12]. Three-dimensional indoor models can be presented in different levels of detail. The level of detail is defined in five levels, from 0 to 5 (Figure 3) [27].

Due to the existing challenges in the indoor environment, the conducted studies usually provide a shell model with or without doors and windows, but in some studies, with the aim to increase the level of detail of the model, openings were modeled and obstacles were approximated as an OBB [14]. There are also studies that focus specifically on door detection in point cloud data [28].

In some previous studies, the methods used were limited to modeling Manhattan space [15,21,29], while in others, there was no limitation and the algorithms were able to model structural elements that were not parallel to the main coordinate axes.

To conclude this section, gaps, noise and complexity of the point cloud, as well as the presence of interior furniture, should be considered as the main challenges of the building interior modeling process, which are still under discussion among researchers [23]. Our main goal in this study is to develop an innovative model-driven method to reduce the effect of the mentioned challenges in providing a solid, watertight 3D model of the interiors of multi-room environments.

## 3. Methodology

In most data-driven methods, the extraction of structural planes is an important step in the reconstruction of a high-quality 3D model. Model-driven methods can be transformed into predefined libraries that are useful resources for modeling different features. In the real world, interiors are built in different ways depending on geographical and cultural conditions. Leaving aside complex non-Manhattan structures and specific modern architecture, the important point is that a wide range of closed spaces can be modeled by combining one or more rectangular shapes.

**Figure 4:**
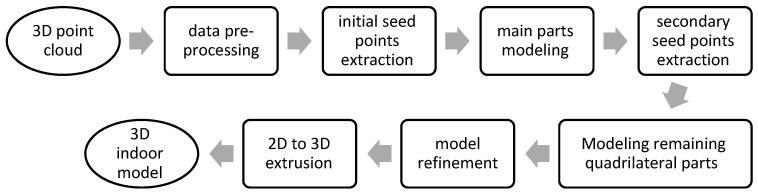
General flow diagram of proposed approach.

The main idea in this study is to grow an initial rectangular model inside each closed space in an XY projection of the point cloud to reach the boundary of each closed space. The general flowchart of the proposed approach is shown in Figure 4.

### 3.1. Data Pre-Processing

The only input required is a 3D point cloud. Some methods require the main walls to be perpendicular or parallel to the X or Y axis of the point cloud [21], which is usually resolved in the registration phase of the point cloud, such as the ISPRS benchmark datasets. However, if for some reason there is no such state in the data by default, then the necessary rotation around the main axis should be estimated to align the point cloud [30]. The point cloud is also shifted to a sharp isolated point to be clear both in the point cloud space and in the occupancy map space. This is conducted to facilitate registration between the point cloud and the occupancy map. Then, the extra scanned parts outside the desired room and stairs should be deleted. Also, if we have a multi-level point cloud, different levels are separated based on the peaks of the height histogram [15].

### 3.2. Initial Seed Points Extraction

**Figure 5:**
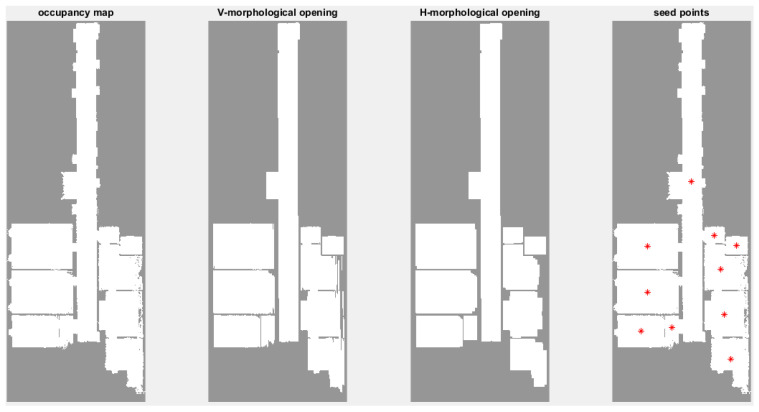
Initial seed points (red dots) extraction procedure from left to right.

In order to model each closed space, separately, a seed point should be inserted in each closed space. To increase automation and avoid user intervention, an approach based on the occupancy map and morphological operators is used [24]. The occupancy map is a point cloud projection in the XY plane with a certain resolution in image space. If there is a point in each pixel, the corresponding pixel value is 1; otherwise, the pixel value is 0. We use 3 to 5 times the average point spacing as the size of the occupancy cells [15]. Also, due to the presence of noise in the point cloud, the pixel size should not be larger than about one third of the thinnest wall to avoid connecting adjacent rooms. The opening morphology operator with a linear structural element with the size of a doorway connecting adjacent rooms is used in two main directions to eliminate the existing connections between the rooms. Then, the connected parts are extracted in 2D space using 2D connected components and the center of mass of each part is considered the initial seed point of this closed space (Figure 5). In this process, spaces smaller than the size of the structural element, such as the bathroom, may be removed, but considering that the modeling is conducted in two stages, the lost small spaces are recovered in the secondary seed point extraction stage. The corridor can also be removed if the width of a corridor is smaller than the doors connecting the rooms.

### 3.3. Model-Driven Modeling

**Figure 6:**
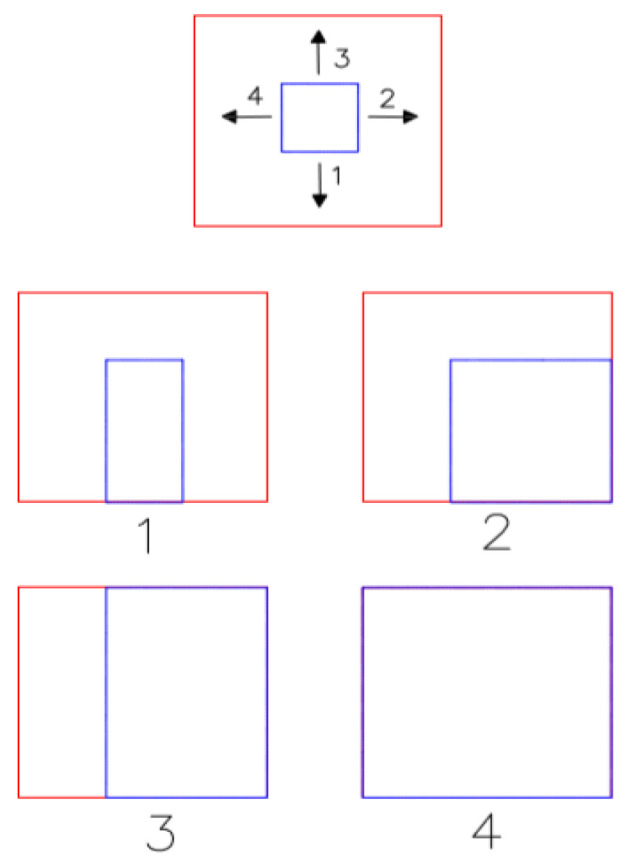
Dynamic graphical presentation of the model-driven idea used in this study.

The initial model starts to grow from the bottom or in a counter-clockwise direction, and when it reaches the boundary of each closed space on each side, it stops growing in that direction. This process is repeated twice to cover different types of spaces; then, the primary and secondary rectangles are combined to reconstruct a 2D model in an XY projection. In Figure 6, the blue rectangle is the initial model and the red one is the ground truth in the XY projection. Also the numbers show the order of model growing procedure.

#### Main Parts Modeling

The input parameters of the modeling function and its performance are examined below.

Point Cloud: The density of the point cloud data is first reduced so that the average distance between points is at least one centimeter to speed up the implementation of point cloud processing. Then, all points are projected onto the XY plane, as the modeling method in this study is projection based.

Occupancy map and seed point coordinates: As mentioned above (cf. Section 3.2), the occupancy map and the coordinates of the seed point are extracted before reducing the density of the point cloud.

Initial model growth rate: The initial model starts to grow from the bottom and in a counter-clockwise direction to find the surrounding walls. The amount of growth in each iteration in metric units is referred to as the initial model growth rate. This parameter should not be greater than half of the thinnest wall. Also, assuming a normal error distribution in the data acquisition step, it should not be less than about two times the standard deviation of the sensor.

Dimensions of the initial model: The initial model in this section is a square. It should be noted that the dimensions of the initial model should not be larger than the actual model, as the initial model only grows in this algorithm. It is sufficient to adjust the dimensions of this square based on the smallest area available in an indoor multi-room complex. A side length of 1 to 1.5 m is suggested for this parameter.

Row and column of the point cloud in the pixel coordinate system: Considering that during the modeling process, the transition between point cloud and occupancy map is needed, this parameter is used for this purpose as mentioned in Equation (1).

Col and row are the pixel coordinates of each point, x_0_ and y_0_ are the origin of the vector coordinate system in the pixel coordinate system and the pixel size is the dimension of each pixel. X and Y are the vector coordinates.

(1) $$\begin{array}{l} X\;=\left(col-x_{0}\right)\times pixel\;size\\ Y\;=\left(y_{0}-row\right)\times pixel\;size \end{array}$$

**Figure 7:**
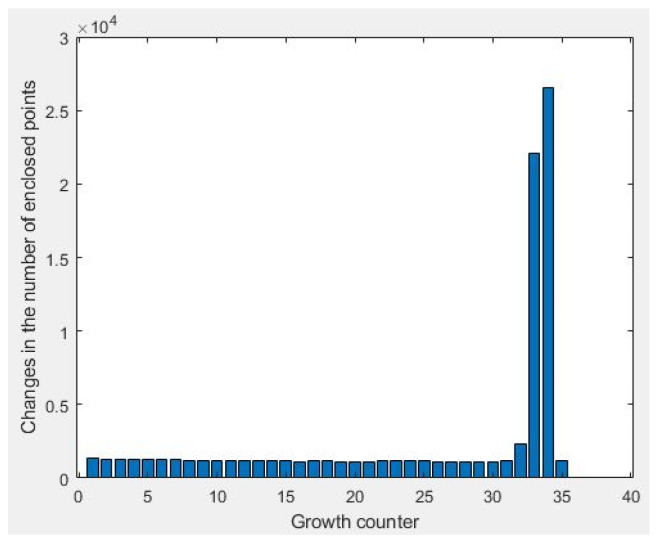
Histogram of changes in number of points enclosed in the rectangle when passing through the boundary of room.

Significant rate of change in the number of points enclosed in the rectangular model (N): The most basic remaining question is how the algorithm detects that it has reached the surrounding walls. After the initial model is created and begins to grow, the number of points enclosed in the initial model are counted at each iteration, and then the model is taken one step further. When a vertical element such as a wall is reached, the number of enclosed points jumps (Figure 7). Considering factors such as the density of the point cloud, possible gaps in the data and the complexity that exists in point cloud data, it is not logical to set a threshold based on the number of points to detect a significant change. Therefore, a change of more than N times of the counted enclosed points compared to previous iterations is considered as an obvious change. N is recommended to be between 2 and 3.

**Figure 8:**
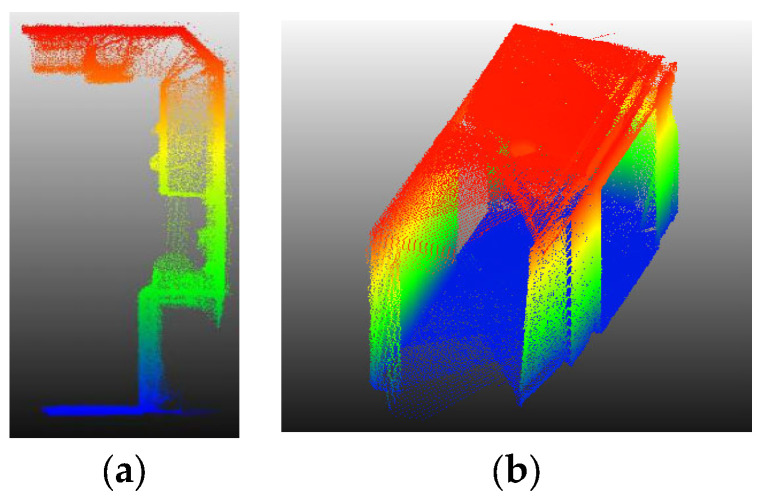
Point cloud challenges to determine the correct stopping condition. (**a**) Priority of interior vertical furniture to the main wall, (**b**) poor points on the entrance of narrow space.

Another issue that is very important is interior furniture. In the internal environment, there are other vertical elements in front of walls. When the initial model reaches them, the number of enclosed points change significantly and comparably to the value of a wall or even more. Examples include a wardrobe, library, cupboard and any other vertical non-wall element. Figure 8a, shown in an XZ projection, shows a growing model that may be erroneously stopped before reaching the correct wall. Thus, in a key sentence, it can be said that the condition “significant rate of change” is a necessary condition, but not a sufficient condition. Therefore, by adding another parameter and its associated condition, this challenge is also met.

The relative amount of data in the arrays after probable wall (R1 and R2): During the model growth process, after each probable wall detected, two linear arrays (rows or columns) equal to the length of the growing side are checked in the occupancy map space. The ratio of white pixels to the total length of each array is stored as *R*1 and *R*2 (Equation (2)).

(2) $$\begin{array}{l} R1=\frac{\mathrm{number}\;\mathrm{of}\;\mathrm{white}\;\mathrm{pixel}\;\mathrm{in}\;\mathrm{the}\;\mathrm{first}\;\mathrm{array}\;\mathrm{after}\;\mathrm{probable}\;\mathrm{wall}}{\mathrm{total}\;\mathrm{length}\;\mathrm{of}\;\mathrm{first}\;\mathrm{array}\;\mathrm{after}\;\mathrm{probable}\;\mathrm{wall}}\\ R2=\frac{\mathrm{number}\;\mathrm{of}\;\mathrm{white}\;\mathrm{pixel}\;\mathrm{in}\;\mathrm{the}\;\sec\mathrm{ond}\;\mathrm{array}\;\mathrm{after}\;\mathrm{probable}\;\mathrm{wall}}{\mathrm{total}\;\mathrm{length}\;\mathrm{of}\;\sec\mathrm{ond}\;\mathrm{array}\;\mathrm{after}\;\mathrm{probable}\;\mathrm{wall}} \end{array}$$

**Figure 9:**
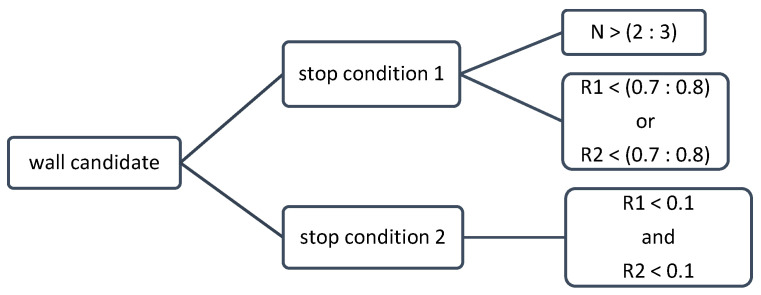
Stop conditions during model growing procedure.

If the most likely element to be detected is interior furniture, the value of these parameters is close to 1 and the model will continue to grow to detect the real wall. However, if this element is a surrounding wall, the value of this relative parameter will be lower and the growth in this direction will be stopped properly. Usually, there are no white pixels in occupancy maps after walls, or there are a few white pixels due to noise, blunders or parts of the connecting port of the rooms. During the growth process, this parameter is calculated iteratively to extend the leading side. Whenever this condition and the previous one are fulfilled at the same time, the growth of the rectangle stops in that direction. A value from 0.7 to 0.8 is recommended for this relative parameter. In addition, if the value of this parameter becomes less than 0.1, the growth in that direction will stop regardless of the first condition (significant rate of change). This stopping condition can be particularly useful at the entrance of narrow corridors with poor data (Figure 8b) due to the glass doors. The stopping conditions are reviewed in Figure 9.

So far, the 2D modeling of the rectangular spaces and the main part of the polygonal spaces has been conducted. After 2D modeling these parts, points inside each model and a buffer equal to half the thinnest existing wall are deleted.

**Figure 10:**
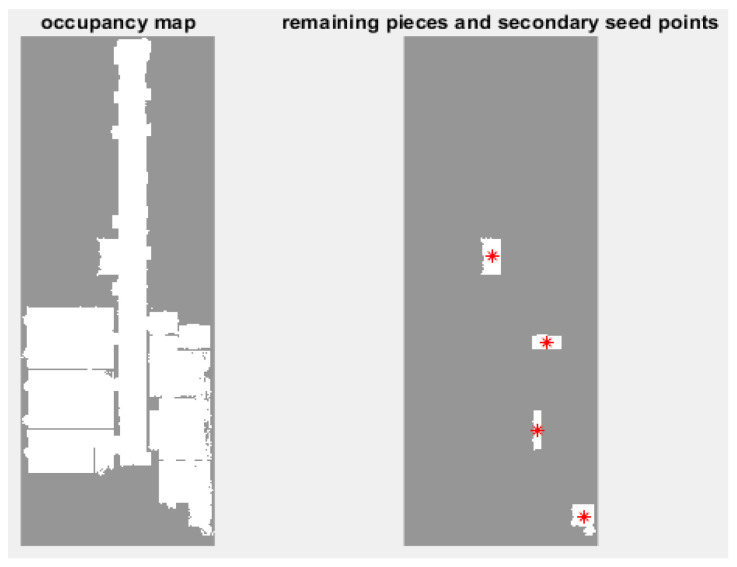
Remaining parts and secondary seed points (red dots) extraction.

Up to this point, rectangular spaces and the main parts of non-rectangular spaces have been modeled; the secondary parts of non-rectangular spaces are still left (Figure 10).

### 3.4. Secondary Seed Points Extraction

To model the remaining parts, we need secondary seed points. The remaining point cloud is converted into a new occupancy map. It may also contain unnecessary small parts, such as part of the door frame. Therefore, the minimum area of a useful part is set to one square meter and other parts are discarded. Then again, the center of mass of each piece is calculated as a secondary seed point.

### 3.5. Modeling Remaining Quadrilateral Parts

At this stage, we use the same process as for modeling the main parts, but with just one more stop condition. Regardless of the number of sides, usually in Manhattan space, the secondary parts are bounded by the walls or one of the previously modeled main parts. In other words, a secondary growing model inevitably leads to a surrounding wall or meets one of the fitted models in the first state. So, in addition to the previous stop conditions, the new stop condition is that, when the intersection of the growing model and the main modeled parts is not empty, the model growth in that direction should be stopped. The fulfilment of this condition is checked like the previous conditions after each repetition in growth process.

**Figure 11:**
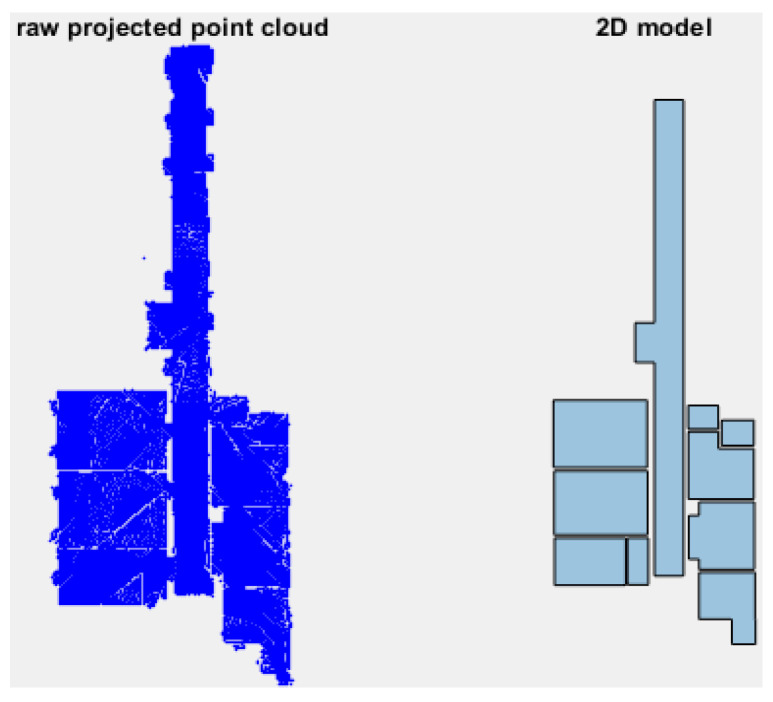
An example of a 2D Manhattan model.

Considering that the secondary parts can have any length and width, the dimensions of the initial model are deliberately set to half the length and half the width of each connected part in the occupancy map. The primary and secondary modeled parts are then merged to reconstruct the 2D Manhattan model (Figure 11).

### 3.6. Model Refinement

Depending on the characteristics of the data, 2D modeling can end in the previous step, but sometimes, a refinement process may be necessary for two reasons. The first is small intrusions that are modeled contrary to reality due to the limitation of stopping conditions during the sequential growth process. The second is problems caused by modeling non-Manhattan spaces with a predefined Manhattan model. These parts are modeled with the aim of increasing the level of detail of the shell model in the XY plane.

**Figure 12:**
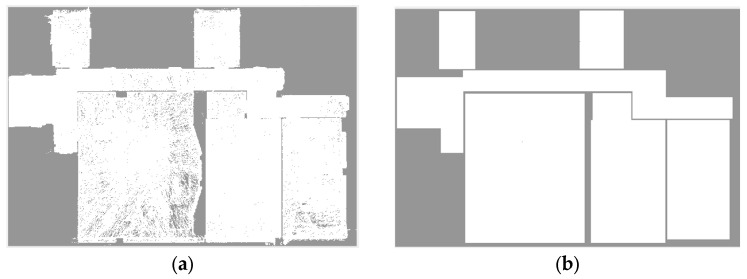

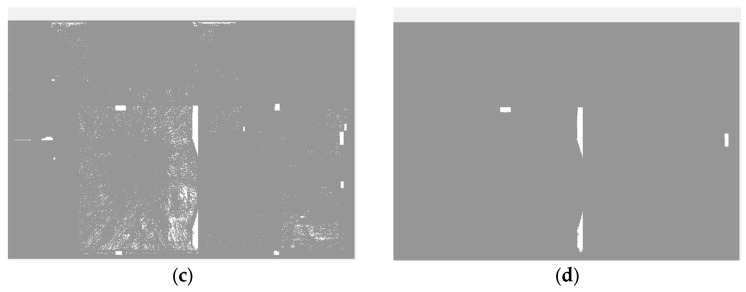
The process of meaningful parts detection, (**a**) occupancy map, (**b**) projected 2D model, (**c**) b-a, (**d**) meaningful parts.

**Figure 13:**
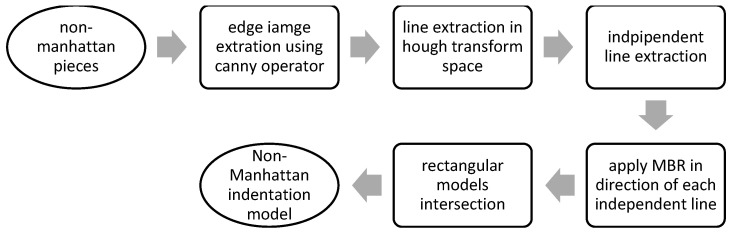
The process of extracting and modeling non-Manhattan intrusions using CMBR.

To solve these problems, a hybrid modification method is used. The reconstructed 2D model is transferred to a new occupancy map, from which the first occupancy map is subtracted. The value of negative pixels is also replaced by zero. There are Manhattan and non-Manhattan intrusions and pixels caused by the insufficient density of point clouds in the difference image. The first and second cases are identified by determining the minimum meaningful area and the third case is ignored (Figure 12). According to environment conditions and point cloud, a minimum meaningful area is chosen either based on the user’s previous knowledge or measurement from the point cloud. Otherwise, this threshold limit can be determined by trial and error. A bounding box is then fitted to each detected part based on the MBR-based method described in [31]. If the ratio of white pixels to total pixels in the box is close to one, the part is rectangular and modeled with the mentioned bounding box. On the other hand, if this ratio is lower than the threshold value (0.8 is recommended based on tests on real-world datasets), this part is located in a non-Manhattan area and therefore the combined MBR (CMBR) [31] has to be used (Figure 13). In this method, based on each orientation, an MBR (rectangle) polygon is estimated as the first approximation level. The intersection of the rectangles corresponding to each orientation produces the approximation of non-rectangular pieces.

**Figure 14:**
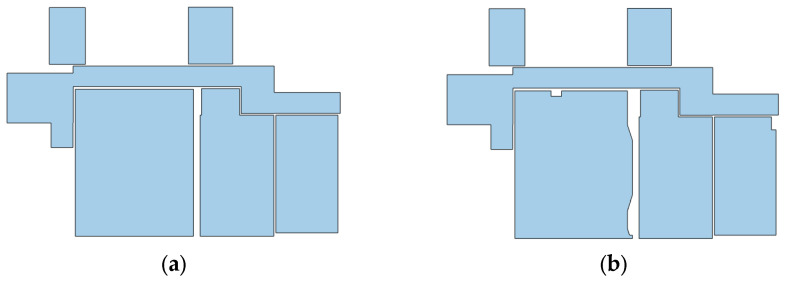
2D models: (**a**) Initial 2D Manhattan model; (**b**) final 2D non-Manhattan model after intrusion removal.

First, the edge image is calculated based on the Canny operator. Lines are extracted using the Hough transform, and then peaks with more than the mean of the Hough image are extracted. Considering that the goal is to extract only independent lines, the group of lines whose theta parameter difference is less than 10 degrees is simplified to the maximum peak of this line group. MBR is applied in the angular direction of each line [31]. The common area between the fitted rectangular pieces is considered a 2D model of the non-Manhattan intrusion piece. Figure 14 shows the results of the modified section.

Finally, the Manhattan and non-Manhattan parts are subtracted from the initial 2D model. It should be noted that there may be narrow parts of the model on the sides of the model because the coordinates of vertices of the initial model and the intrusions do not necessarily match exactly. Therefore, coordinates of vertices of intrusion that are less than twice the pixel size of the binary image from the initial model are converted to the coordinates of the vertices of the initial model.

### 3.7. 2D to 3D Extrusion

Using the height histogram is a classic solution for ceiling and floor extraction [8]. Considering that 2D vertices of each space have been determined, the peak of the z-histogram of the point cloud is calculated for each closed space. Then, the maximum frequency of the first quadrant (left side) is extracted as the floor plane and the maximum frequency of the last quadrant (right side) is extracted as the ceiling plane of each closed space. Finally, to present the 3D model, the 2D model is automatically extruded into a 3D model based on the extracted floor and ceiling.

## 4. Experiment and Results

The proposed approach was implemented in MATLAB^®^ on a personal notebook (i7-7500U CPU, GeForce 940mx GPU, with 8 GB memory). The capabilities of the projection module of CloudCompare (https://www.danielgm.net/cc/, accessed on 1 August 2022) were also used to generate the occupancy maps.

The proposed approach was evaluated on the five ISPRS benchmark datasets, including TUB1, TUB2 first floor, TUB2 second floor, UVigo and UoM based on the evaluation method presented in [32]. The specifications of the ISPRS datasets used for the evaluation are shown in Table 1.

There are source model (S) and ground truth model called reference model (R) in the literature [32]. Also, b is a buffer with specific dimensions around the reference model.

Completeness: according to Equation (3), the numerator of the fraction is the sum of the projected common area between the source model surfaces and a buffer with a certain size around the reference model. Also, the denominator of the fraction is the sum of the surface areas of the reference model.

(3) $$M_{comp}(S,R,b)=\frac{\sum_{i=1}^{n}\sum_{j=1}^{m}\left|S^{i}\cap b(R^{j})\right|}{\sum_{j=1}^{m}\left|R^{j}\right|}$$

Correctness: According to Equation (4), the numerator of the fraction is the sum of projected common area between the source model surfaces and a buffer with a certain size around the reference model. Also, the denominator of the fraction is the sum of the surface areas of the source model.

(4) $$M_{corr}(S,R,b)=\frac{\sum_{i=1}^{n}\sum_{j=1}^{m}\left|S^{i}\cap b(R^{j})\right|}{\sum_{i=1}^{n}\left|S^{j}\right|}$$

Geometric accuracy: In the accuracy computation, the reference is represented by applying a uniform space sampling on the surfaces of the 3D reference model. According to Equation (5), accuracy is defined as the median orthogonal distance between the resampled points (*p_i_*) and the closest surfaces of the source model (*π*_*j*_). Cut-off distance r is defined as the maximum acceptable distance between a reference point and the closet source surface. The distance greater than r is ignored in calculating the geometric accuracy.

(5) $$M_{Acc}(S,R,r)=med\left\|\pi_{j}^{T}p_{i}\right\|,if\left\|\pi_{j}^{T}p_{i}\right\|\leq r$$

**Figure 15:**
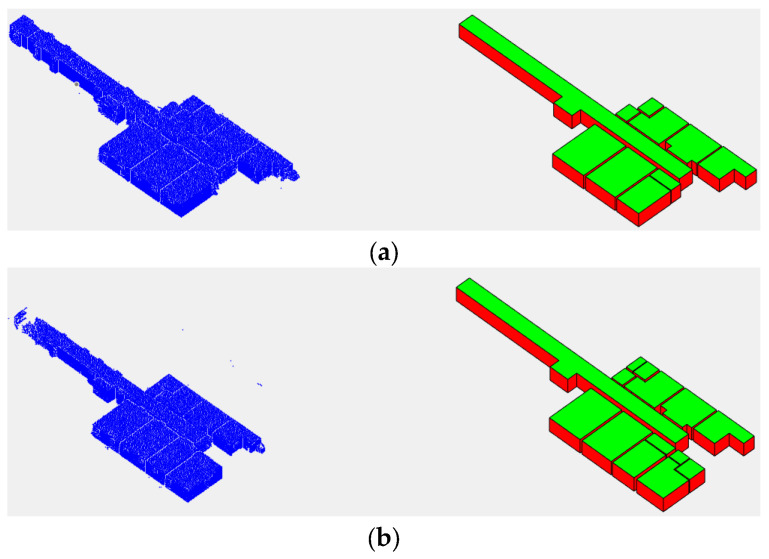

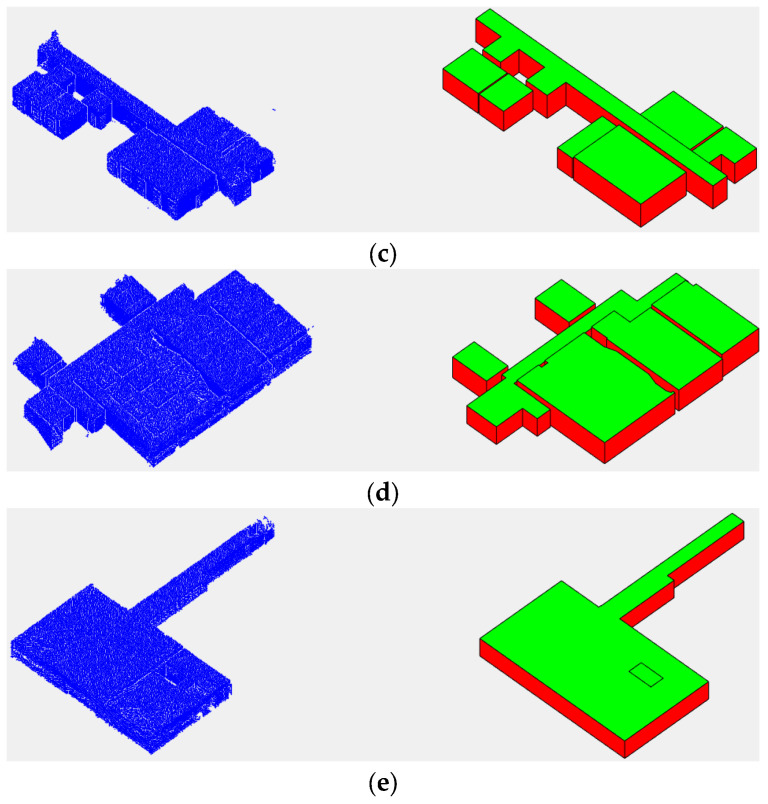
The ISPRS indoor modeling benchmark point clouds and 3D reconstructed models by proposed approach: (**a**) TUB1, (**b**) TUB2-first floor, (**c**) TUB2-second floor, (**d**) UoM and (**e**) UVigo.

The result of the reconstructed 3D model together with the 3D point cloud of each data point is shown in Figure 15. The whole modeling process took 230, 170, 98, 122 and 101 s for each dataset from (a) to (e), respectively, shown in Figure 15. Processing time, point cloud data loading time and time required by the user to determine input parameters are not included in this time. Visually, all the reconstructed models, especially in the case of TUB1 and TUB2, appear very suitable according to their point clouds and the reference model in IFC format. In TUB1 and TUB2, all existing walls were identified and no additional walls were reconstructed. The main reason can be considered to be the specification of the initial model according to the geometry of the space in the proposed model-driven approach. Also, the application of the effective stopping condition in the model growing step in the case of Manhattan environments can be mentioned. However, there are some unwanted extra parts in UoM and some missing elements in UVigo. In the case of UoM, the main reason is the non-fulfilment of the second condition due to the presence of a lot of noise between the two adjacent walls. In the case of the UVigo, due to the presence of many details in the essence of the data, high-amount clutters in point clouds and multi-height ceilings, the proposed model-driven method is not able to model all these details. These mentioned cases can be considered the limitations of the algorithm; if necessary, the model must be corrected manually.

**Figure 16:**
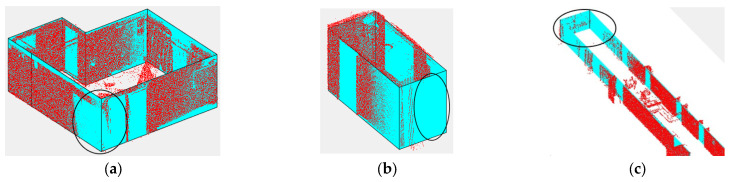
Examples of the ability of the proposed method to deal with point could gaps caused by (**a**) open doors, (**b**) Unscannable materials or obstacles, (**c**) Unscannable materials or obstacles.

As mentioned, one of the main goals of the study was to manage gaps in point clouds in the 3D modeling of interior spaces. In Figure 16, some examples of success of the proposed method in dealing with this challenge can be examined.

Also, the values of completeness, correctness and geometric accuracy are calculated in a 10 cm buffer to be comparable with the results obtained and available from previous studies in similar conditions [32]. This is achieved manually using predefined AutoCAD functions and abilities.

**Figure 17:**
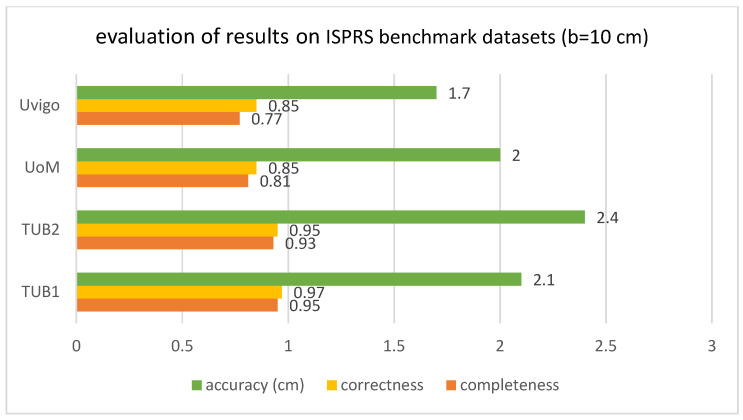
Results evaluation on ISPRS benchmark datasets (b = 10 cm).

**Figure 18:**
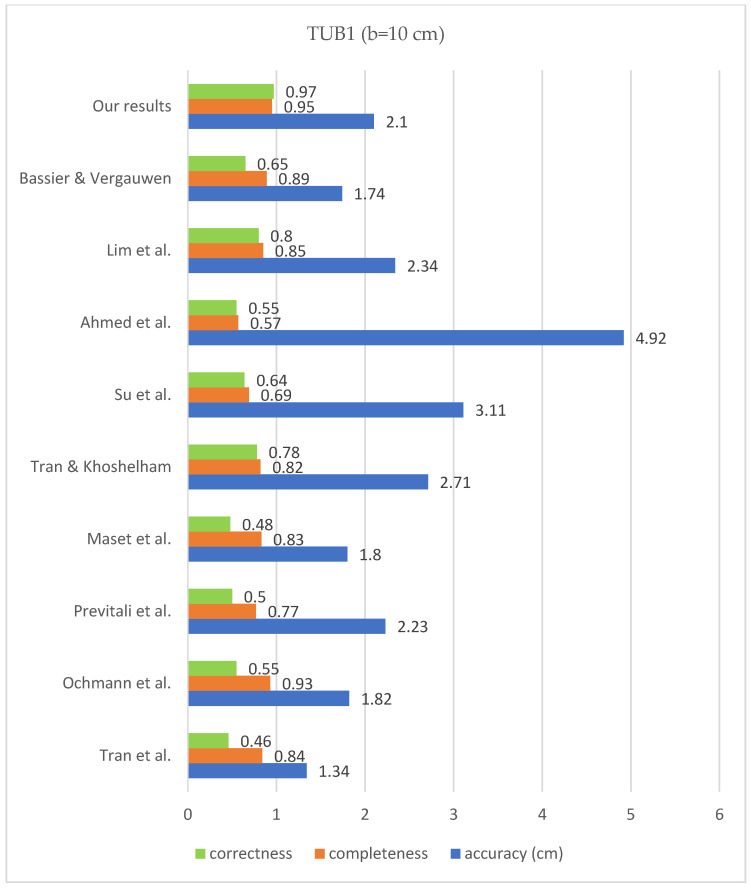
Results comparison for TUB1; Bassier & Vergauwen [20]; Lim et al. [17]; Ahmed et al. [18]; Su et al. [19]; Tran & Khoshelham [2]; Maset et al. [33]; Previtali et al. [13]; Ochmann et al. [16]; Tran et al. [21].

**Figure 19:**
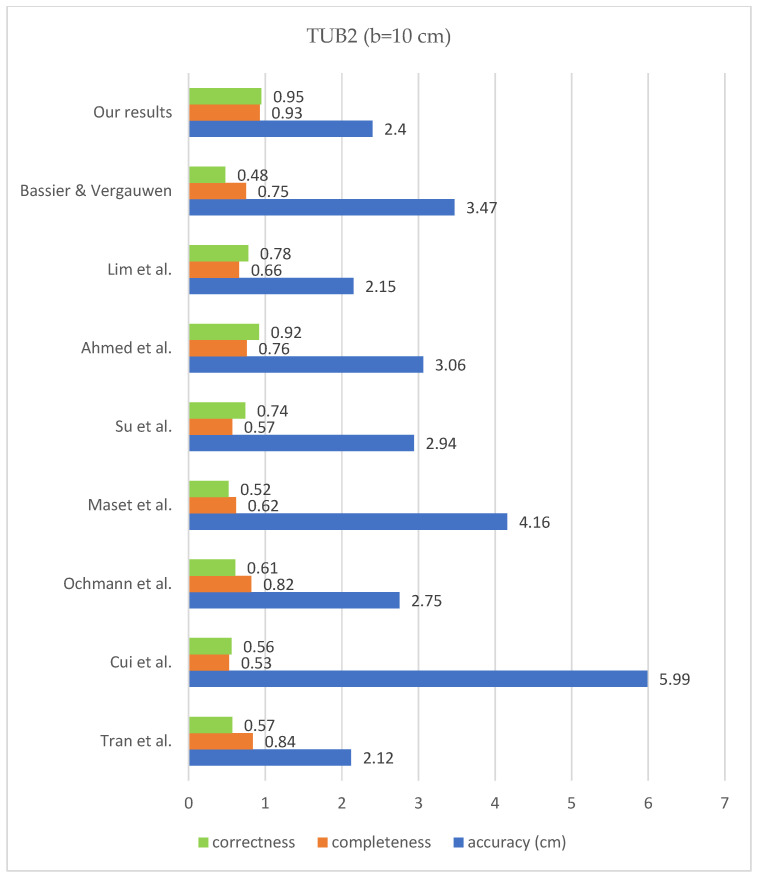
Results comparison for TUB2; Bassier & Vergauwen [20]; Lim et al. [17]; Ahmed et al. [18]; Su et al. [19]; Maset et al. [33]; Ochmann et al. [16]; Cui et al. [34]; Tran et al. [21].

**Figure 20:**
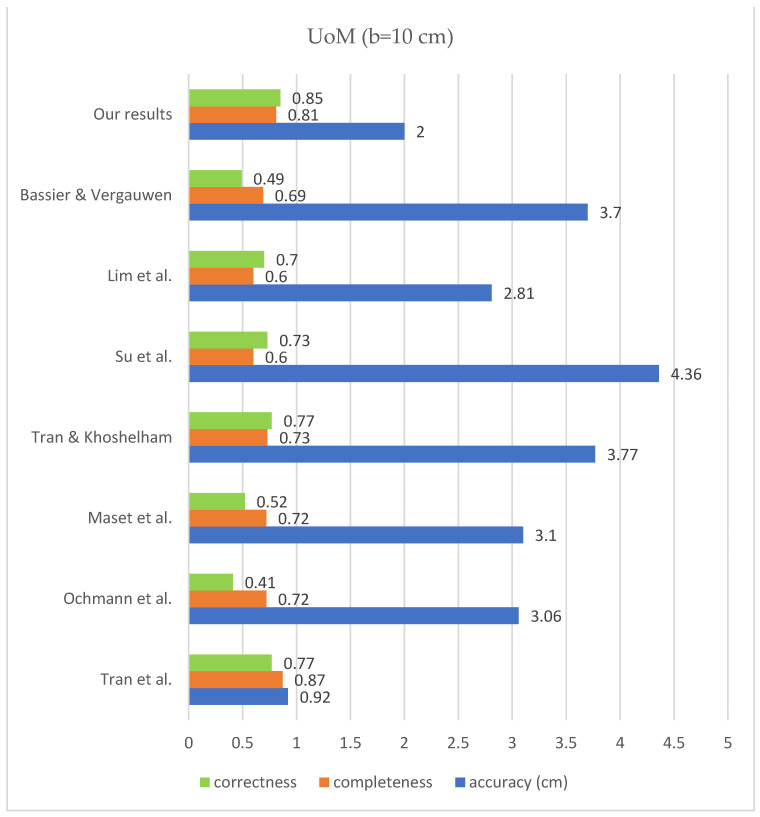
Results comparison for UoM; Bassier & Vergauwen [20]; Lim et al. [17]; Su et al. [19]; Tran & Khoshelham [2]; Maset et al. [33]; Ochmann et al. [16]; Tran et al. [21].

**Figure 21:**
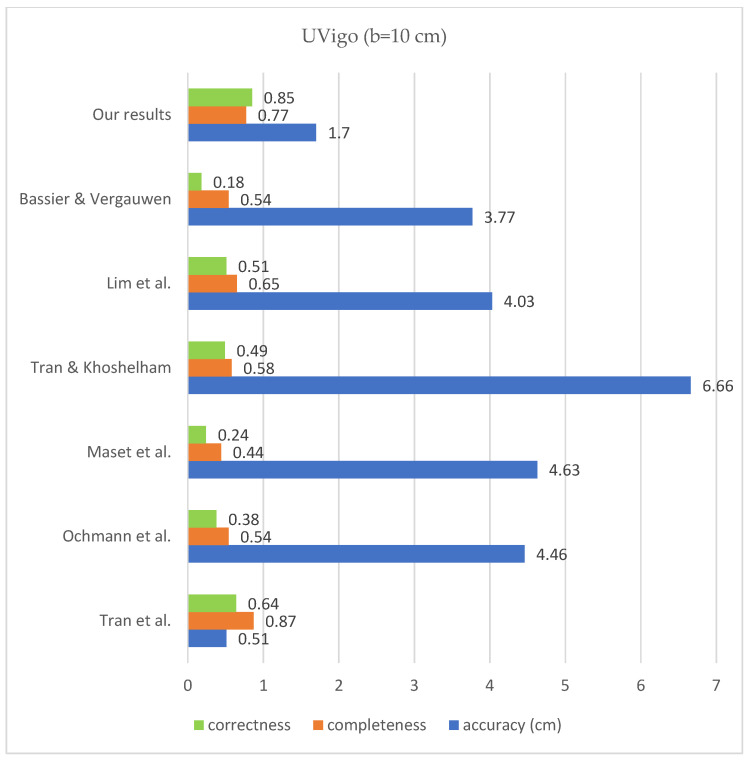
Results comparison for UVigo Bassier & Vergauwen [20]; Lim et al. [17]; Tran & Khoshelham [2]; Maset et al. [33]; Ochmann et al. [16]; Tran et al. [21].

According to the bar chart below (Figure 17), the correctness improved compared to the previous state of the art on all mentioned datasets, according to the research of [32]. The level of completeness also improved for the data of TUB1 and TUB2 in comparison with previous works, and it is in the second place in the case of UoM and UVigo. In addition, the level of accuracy is close to the median of the previous successful works. The values of completeness, correctness and geometric accuracy are compared with some previous studies on the mentioned datasets and under the same conditions in Figure 18, Figure 19, Figure 20 and Figure 21.

The determination of an initial model corresponding to the geometry of the space, which is the general framework of the model-driven modeling process, is the main reason for the significant completeness and correctness values obtained. In other words, the modeling of spaces with more or fewer sides or false intersections of surfaces is avoided by initializing the model according to the geometry of each space. Among other reasons, we can also mention the remarkable efficiency of the stopping conditions used during the model growing. They are consistent the characteristics of the point cloud data.

## 5. Conclusions and Future Work

In this paper, 3D modeling with a model-driven active shape is implemented for the first time in the field of interior modeling, to our best knowledge. The values of completeness, correctness and geometric accuracy prove the ability of the algorithm to deal with local gaps, clutter, complexity and the interior furniture of the point cloud as the main issues of the interior modeling. One of the main benefits of our method is to consider an initial model appropriate to the usual data geometry, and also the use of robust stopping conditions. Results successfully indicate that the algorithm has the ability to achieve the mentioned goals, namely, dealing with the local gaps, noise, clutter and complexity of point clouds. Additionally, on account of the computational simplicity of the presented algorithm and also the time of the procedure, the algorithm has computational and time efficiency.

However, the over/under segmentation in the seed point extraction step can be mentioned as a limitation of the proposed method. It is a consequence of the effect of point cloud with an inappropriate density, which causes discontinuity in the occupancy map. If it occurs, it can be solved by trying to tune the structural element size. Also, according to the stopping condition of the model growth step, one of the challenges is the sides of a closed space, the main surface of which is the empty space caused by the door. This can lead to faulty joints in adjacent tight spaces. Some spaces are identified as partial Manhattan spaces (UoM, UVigo). As mentioned, these spaces can be modeled with a “model refinement” section using CMBR. However, if we consider datasets that are absolutely non-Manhattan, it cannot be modeled with our proposed method. The quantity and quality of reconstructed components directly depend on the point cloud conditions and density in the model refinement step. The proposed approach does not cover uneven roofs, doors and windows, or curved elements.

The development of this method as well as the presentation of other innovative model-driven methods seems to be valuable according to the results of this study. One of the future works could be to expand the modeling library from rectangular shape to more complex basic shapes to model different types of room architecture. To this end, multi-level model-driven 3D modeling could be an interesting research direction. For this, basic shapes like rectangles, triangles and circles or arcs could be considered, and their combination to build more complex structures as the components of the next higher level could be implemented. Also, as a prerequisite for development of such model-driven methods, the reliable separation of closed spaces based on segmentation concepts can be another field of future works.

## Figures and Tables

**Table 1:** Specifications of ISPRS datasets used for evaluation [32].

Dataset	Sensor	Number of Point (Million)	Mean Point Spacing (cm)	Sensor Trajectory	Clutter	Multi-Story	Manhattan World
TUB1	Viametris IMS3D	33.6	0.5	Yes	Low	No	Yes
TUB2	Zeb Revo	21.6	0.8	Yes	Low	Yes	Yes
UVigo	UVigo backpack	14.9	1.0	Yes	Moderate	No	Yes *
UoM	Zeb 1	13.9	0.7	No	Moderate	No	Yes *

* partial deviation from Manhattan world configuration.

## Data Availability

The final results (3D models) obtained in this study are available on request from the first author.
